# Radial Head Arthroplasty Through a Posterior Elbow Approach in Monteggia Type IID Fracture-Dislocation: A Case Report

**DOI:** 10.7759/cureus.64745

**Published:** 2024-07-17

**Authors:** Raphael De Sá Vasconcelos Uchôa, Ana Luiza Simões de Brito Uchôa

**Affiliations:** 1 Traumatology, Agreste Regional Hospital, Caruaru, BRA

**Keywords:** functional outcome after elbow fracture, elbow trauma, adult, treatment result, forearm injuries

## Abstract

Monteggia fracture-dislocation is an inherently unstable injury in the elbow region, characterized by a fracture of the proximal ulna associated with dislocation of the radial head, often with high rates of postoperative complications. Some variants of this injury involve a combination with a fracture of the radial head, which further complicates the scenario, often requiring multiple surgical approaches, increasing soft tissue damage, and promoting a higher risk of complications. The objective of this study is to report the case of a patient undergoing surgical intervention through a posterior approach to the elbow, during which radial head arthroplasty and ulna osteosynthesis were performed. Ambulatory follow-up revealed, through the excellent early functional outcome presented, that minimizing damage through treatment with a single approach provides significant benefits.

## Introduction

Monteggia fracture-dislocation involves proximal ulna fractures associated with radial head dislocation. Bado classified it into the following: I, proximal ulna fracture associated with anterior displacement of the radial head; II, posteriorly displaced radial head; III, lateral displacement; and IV, association with fracture of the proximal one-third of the radius. Jupiter subdivided Bado's type II into the following: IIA, ulna fracture distal to the olecranon and coronoid process; IIB, metaphyseal fracture without involvement of the coronoid; IIC, diaphyseal fractures; and IID, fracture of the proximal one-third of the ulna involving the coronoid and olecranon [1-3]. In Bado's type II, there is an association with radial head fracture in 35% to 100% of cases [1-3]. Monteggia's injury requires surgical treatment, using a posterior elbow approach for open reduction and internal fixation of the proximal ulna [4]. Radial head fractures, on the other hand, should always be evaluated, possibly requiring fixation, excision, or arthroplasty, which necessitates an additional lateral elbow approach, causing greater structural damage [5,6].

## Case presentation

A 67-year-old female patient presented with pain, deformity, and functional limitation in the right elbow following a fall from her own height. X-rays revealed a fracture of the proximal one-third of the ulna involving the olecranon and coronoid processes, associated with posterior displacement of the radial head, with multifragmented fracture of the latter. The diagnosis of Monteggia fracture-dislocation was classified within the Bado type II classification and subtype D by Jupiter's modification of the Bado type II classification.

**Figure 1:**
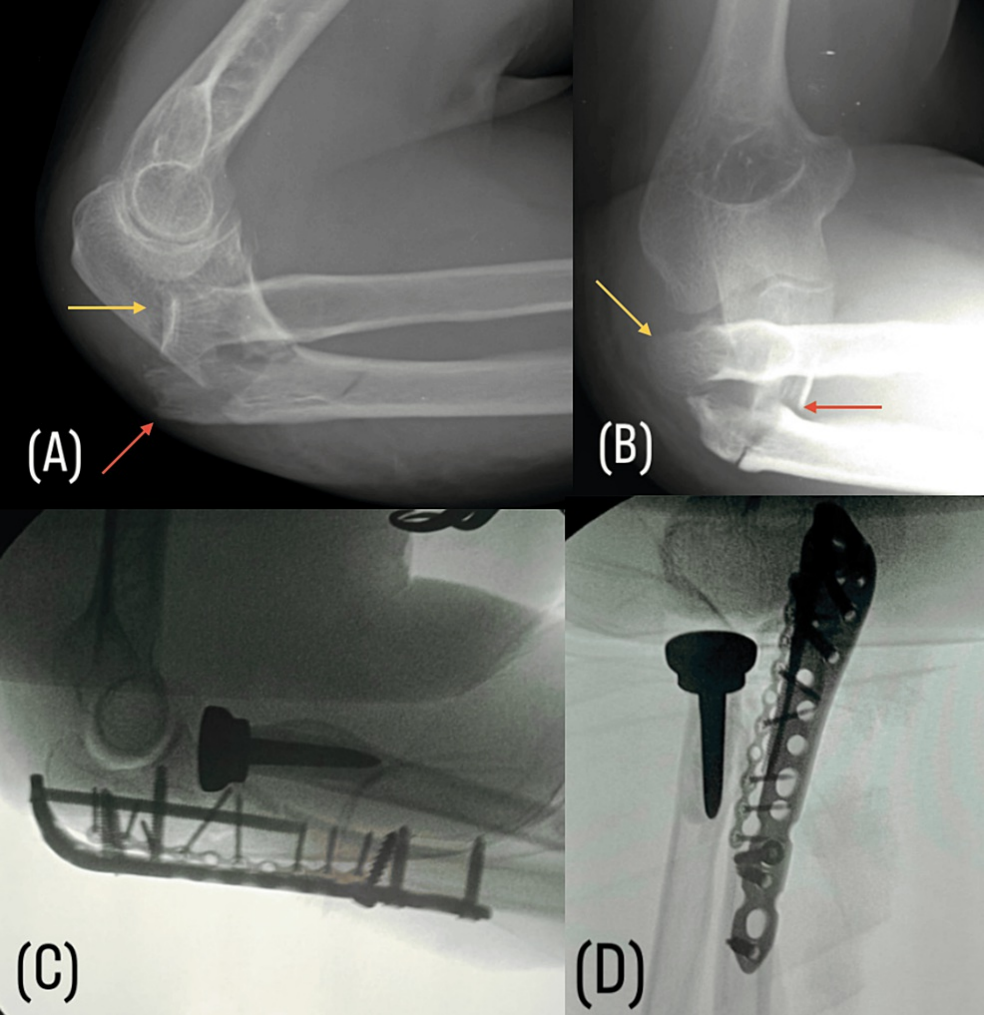
(A, B) Preoperative lateral radiograph showing Monteggia IID fracture-dislocation: fracture of the proximal one-third of the ulna involving the coronoid and olecranon (red arrow) in association with posterior displacement and fracture of the radial head (yellow arrow). (C, D) Intraoperative control images with fluoroscopy.

Initial management opted for definitive surgical treatment with radial head arthroplasty, followed by anatomical reduction of the proximal ulna and fixation using 2.0mm microfragment plates and a locked anatomical olecranon plate. For this purpose, a posterior elbow approach was used to perform all stages of the procedure (Figure 1).

After the ulna synthesis, with the surgical wound still open, elbow range of motion excursion testing was performed in all planes, with direct visualization confirming the restored stability of anatomical and biomechanical relationships. It was decided not to immobilize the limb in the immediate postoperative period, and after hospital discharge, the patient was encouraged to start physical therapy immediately.

**Figure 2:**
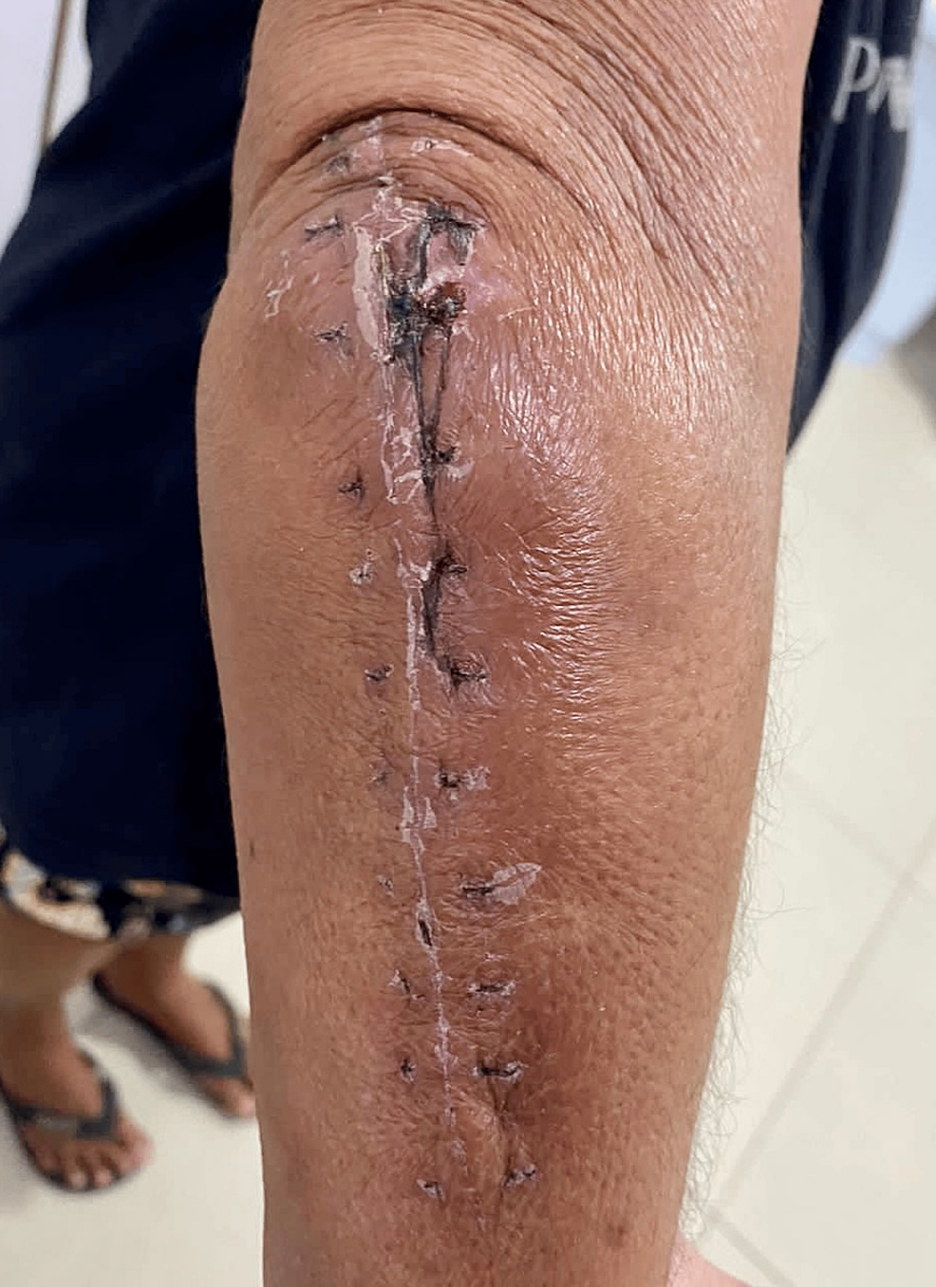
Appearance of the surgical wound on the 15th postoperative day.

**Figure 3:**
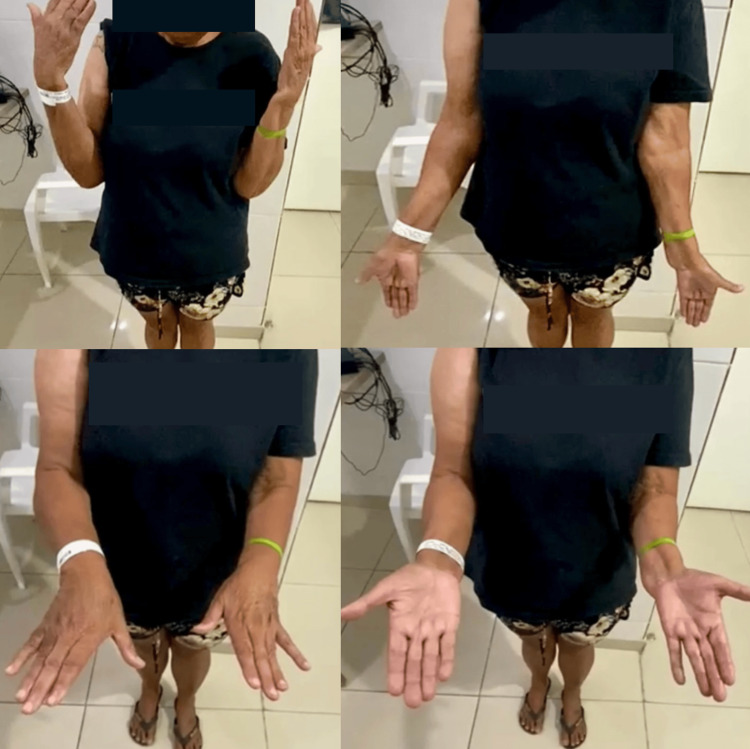
Full range of motion on the 15th postoperative day.

The first follow-up occurred on the 15th postoperative day, when the patient showed excellent progress, without complaints of pain or other complications, satisfactory scar appearance at the surgical wound without secretions (Figure 2), and full range of motion of the right elbow, identical to the contralateral side, and no pain (Figure 3).

Surgical technique

With the patient in a prone position, under brachial plexus block and sedation, the shoulder was abducted at 90° and supported on a cushion at the level of the tourniquet, with the elbow flexed at 90° and the forearm hanging laterally on the table. A longitudinal posterior incision was made in the midline of the elbow region, with slight lateral curvature, avoiding the tip of the olecranon. After visualizing the comminuted fracture focus of the proximal ulna, the upper limb was externally rotated and the elbow maximally flexed to increase deformity, separating the fragments of the proximal ulna and exposing the head and neck of the radius through the approach. Integrity of the annular ligament and lateral ligament complex of the elbow was observed, as well as multifragmented fracture of the radial head. Using an oscillating saw, the remaining intact portion of the radial head was excised, which was used to estimate the size of the prosthesis, and removal of small free fragments was performed. Subsequently, arthroplasty and reduction of the radiocapitellar joint were performed.

**Figure 4:**
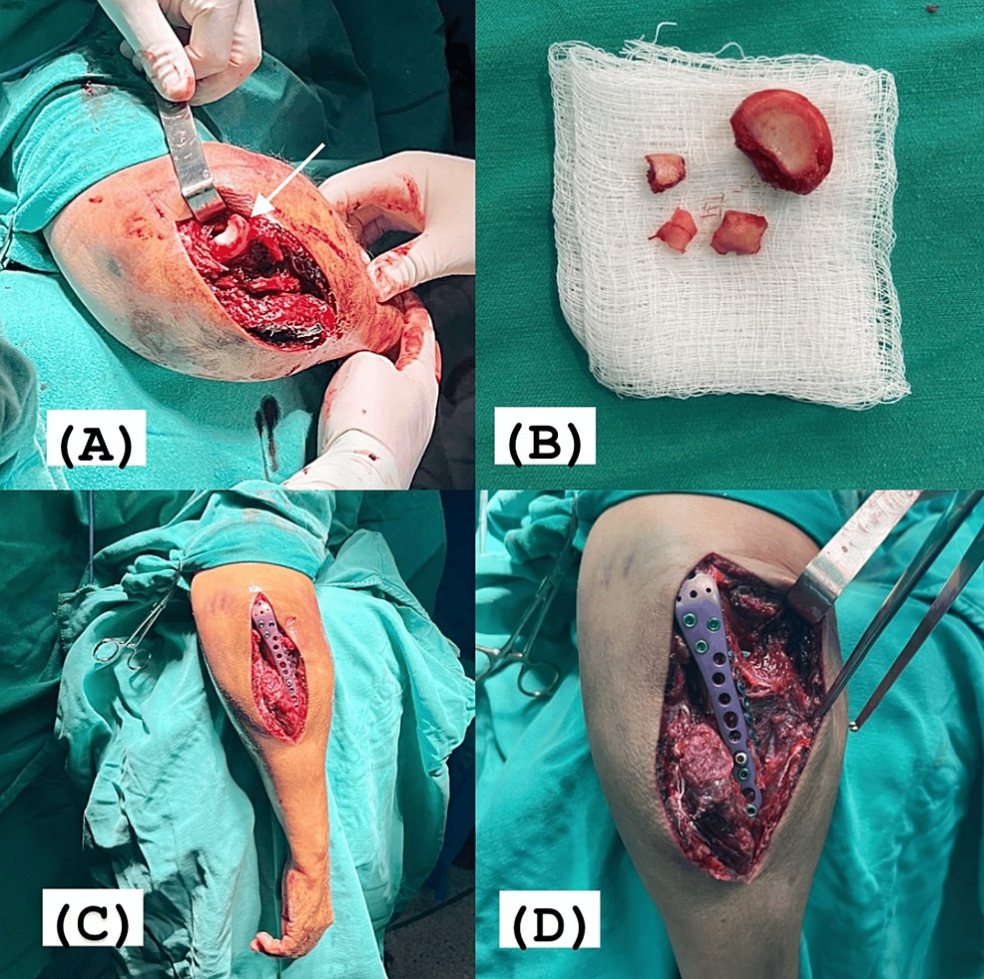
(A) Maximum flexion of the elbow, exposing the head of the radius through the posterior access to the elbow (white arrow). (B) Radial head after excision, demonstrating a comminuted fracture in four fragments. (C, D) Posterior access to the elbow for arthroplasty of the radial head and osteosynthesis of the proximal ulna.

Returning to the initial position, reduction of the small fragments of the proximal ulna was performed, followed by osteosynthesis with 2.0mm microfragment plates and a locked anatomical olecranon plate. Upon completion, the reduction and implant positioning were confirmed under fluoroscopy, with satisfactory biomechanical stability tests (Figure 4).

After releasing the tourniquet, hemostasis was reviewed and closure was performed layer by layer. A compressive dressing with sterile orthopedic cotton and 15cm bandages was applied and maintained for the first seven days. Elbow joint mobilization began on the first day.

## Discussion

Bado type II, a fracture with posterior apex of the proximal ulna and posterior displacement of the radial head, is the most common in adults. Managing these inherently unstable injuries represents a significant challenge, which may be further complicated by the association with radial head fracture [1-3]. Monteggia fracture-dislocation is often associated with poor functional outcomes and high complication rates (27% to 41%), such as residual instability, neurological injuries, heterotopic ossification, nonunion, hardware failure, and joint stiffness [4, 7]. Hence, there is a clear need for a surgical approach aimed at minimizing additional damage and enabling early rehabilitation.

In the literature, the transolecranon approach has been described for Monteggia fractures to repair combined radial head injuries, reducing soft tissue damage and avoiding the need for additional surgical approaches. This approach reduces the complication rate and optimizes functional outcomes [8]. However, despite the stability observed after osteosynthesis, postoperative care protocols typically involved elbow immobilization [4,9,10].

**Figure 5:**
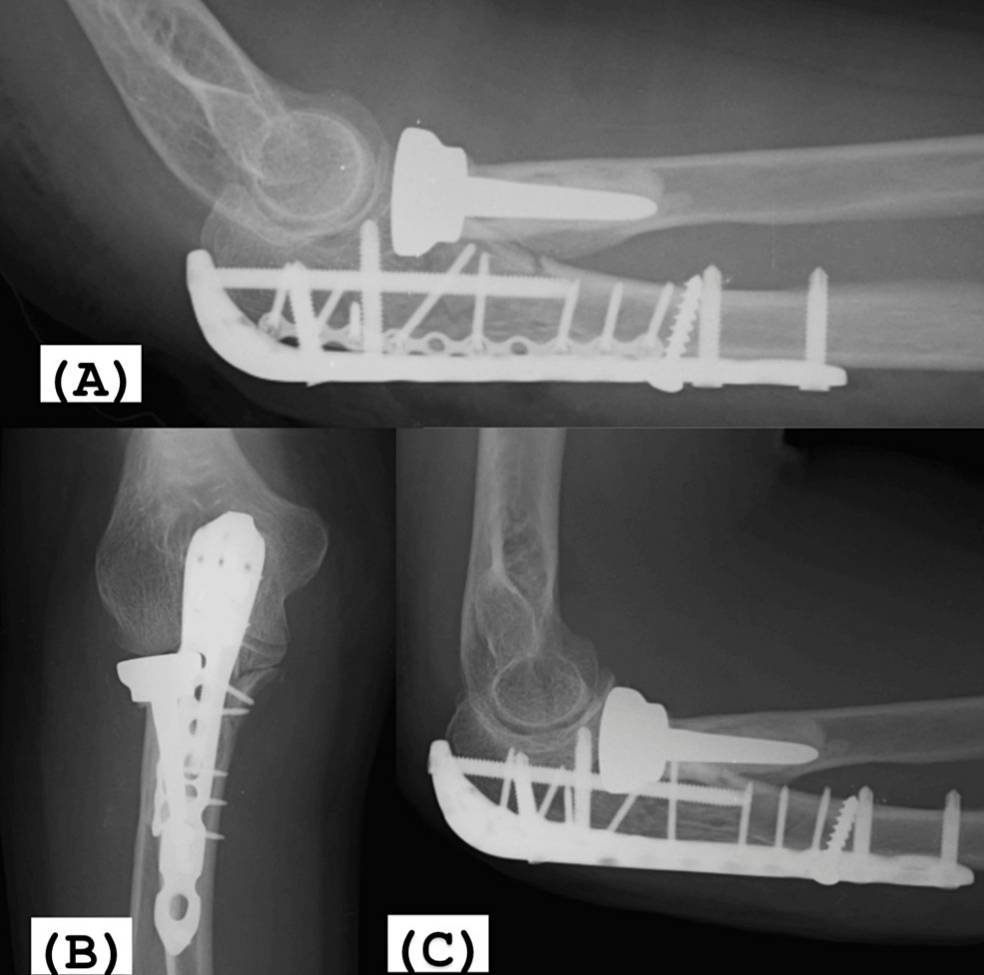
(A) Radiographic control of the immediate postoperative period. (B, C) 15 days postoperatively, fracture reduction and joint congruity were maintained.

In the presented case, approaching the radial head fracture through the ulnar fracture focus allowed for extensive exposure of the deep lateral aspect of the elbow without the need for additional access. This not only facilitated arthroplasty, reducing surgical time, but also preserved soft tissues. Additionally, rigid synthesis with anatomical reduction of the proximal ulna resulted in excellent biomechanical outcomes, with no residual instability observed at the end of structural repair. Consequently, postoperative immobilization was excluded, with only a sling used for comfort. The patient was encouraged to actively mobilize the operated limb the day after surgery and to start physiotherapy immediately postoperatively (Figure 5).

## Conclusions

This protocol demonstrated significant benefits with early recovery of range of motion equivalent to the healthy contralateral limb, with a clear positive impact on functional rehabilitation and no alteration in bone fragment reduction, joint congruence loss, or implant displacement.
